# Measuring the short‐term substrate utilization response to high‐carbohydrate and high‐fat meals in the whole‐body indirect calorimeter

**DOI:** 10.14814/phy2.12835

**Published:** 2016-06-28

**Authors:** Andrei Gribok, Jayme L. Leger, Michelle Stevens, Reed Hoyt, Mark Buller, William Rumpler

**Affiliations:** ^1^ Food Components and Health LaboratoryBeltsville Human Nutrition Research CenterAgricultural Research ServiceUnited States Department of AgricultureBeltsvilleMaryland; ^2^ Biophysics and Biomedical Modeling DivisionUS Army Research Institute of Environmental MedicineNatickMassachusetts

**Keywords:** Indirect calorimetry, metabolic flexibility, regularization

## Abstract

The paper demonstrates that minute‐to‐minute metabolic response to meals with different macronutrient content can be measured and discerned in the whole‐body indirect calorimeter. The ability to discriminate between high‐carbohydrate and high‐fat meals is achieved by applying a modified regularization technique with additional constraints imposed on oxygen consumption rate. These additional constraints reduce the differences in accuracy between the oxygen and carbon dioxide analyzers. The modified technique was applied to 63 calorimeter sessions that were each 24 h long. The data were collected from 16 healthy volunteers (eight males, eight females, aged 22–35 years). Each volunteer performed four 24‐h long calorimeter sessions. At each session, they received one of four treatment combinations involving exercise (high or low intensity) and diet (a high‐fat or high‐carbohydrate shake for lunch). One volunteer did not complete all four assignments, which brought the total number of sessions to 63 instead of 64. During the 24‐h stay in the calorimeter, subjects wore a continuous glucose monitoring system, which was used as a benchmark for subject's postprandial glycemic response. The minute‐by‐minute respiratory exchange ratio (RER) data showed excellent agreement with concurrent subcutaneous glucose concentrations in postprandial state. The averaged minute‐to‐minute RER response to the high‐carbohydrate shake was significantly different from the response to high‐fat shake. Also, postprandial RER slopes were significantly different for two dietary treatments. The results show that whole‐body respiration calorimeters can be utilized as tools to study short‐term kinetics of substrate oxidation in humans.

## Introduction

The recent surge of interest in metabolic flexibility (MF) and its quantification (Kelley and Mandarino 2000; Galgani et al. 2008; Sparks et al. 2009a; Stull et al. 2010; Battaglia et al. 2012; Dube et al. 2014; Kahlhofer et al. 2014; Prior et al. 2014) revitalized attention to the problem of short‐term measurements of substrate oxidation and energy expenditure in humans. Metabolic flexibility was brought into focus after the metabolic inflexibility of oxidative fuel selection has been linked to obesity, insulin resistance, and type 2 diabetes (Kelley and Mandarino 2000; Galgani et al. 2008). Metabolic flexibility is usually measured as hour‐to‐hour fluctuations of the respiratory quotient (RQ) in skeletal muscle or, at a whole‐body level, as day‐to‐day difference in RQ or respiratory exchange ratio (RER) (Galgani et al. 2008). While these long‐term measurements can capture only linear variations in substrate oxidation, the short‐term RQ/RER kinetics can reveal nonlinear dependences and offer new insights into short‐term oxidative dynamics in humans. Since indirect calorimetry remains the “gold” standard to study metabolism, several new techniques have been proposed to calculate instantaneous gas exchange rates obtained in metabolic chambers (Ortigues et al. 1997; Nguyen et al. 2003; Granato et al. 2004; Tokuyama et al. 2007, 2009; Brychta et al. 2009; Gribok et al. 2013). All published methods demonstrated their ability to recover short‐term dynamics of gaseous exchange rates such as rates of oxygen consumption or carbon dioxide production; however, none were proven to be a reliable tool for recovery of short‐term substrate oxidation data such as RER. The difficulty of recovering substrate oxidation kinetics has two facets: first, the RER is a ratio of two variables and thus its accuracy depends not only on the uncertainty in the gaseous exchange rates but also on their absolute values (Taylor 1997; Gribok et al. 2013), making it more difficult to estimate RER for low‐level activities and during sleep. Second, the accuracy of oxygen and carbon dioxide concentration sensors used in the mass spectrometers differ greatly. Differences can be as high as 30 times, with carbon dioxide device being the more accurate analyzer. The difference emerges from the different dynamic range of two gasses that need to be measured.

The availability of a relatively simple analytical tool to measure minute‐to‐minute substrate oxidation would greatly enhance our ability to study key mechanisms of metabolic flexibility such as the rate of change in substrate oxidation, its response to different macronutrients, and ultimately its short‐term kinetics. Having accurate substrate oxidation time series data would also provide an opportunity to analyze metabolic flexibility in the frequency domain, like it is currently performed for heart rate variability and continuous glucose signals. While metabolic response to meals is of great importance, the short‐term response to different exercise challenges would be of great interest also, since along with the caloric intake, physical activity is the second most important factor in metabolic regulation, weight gain/loss, and obesity.

Even though literature mostly agrees on what metabolic flexibility is, the opinions on how to measure it vary quite widely. According to Kelley and Mandarino (2000), metabolic flexibility is the ability of an organism to switch “oxidative energy sources” in response to insulin stimulation or exercise challenge. However, the majority of papers on MF (Ukropcova et al. 2007; Sparks et al. 2009a; Meex et al. 2010) examine ΔRQ (insulin‐stimulated RQ – fasting RQ) as an index of MF. While being a valid measure of the change in substrate oxidation, the index tells nothing about the rate of change in substrate oxidation. As an example, heart rate, whether it is 200 bpm or 60 bpm, is an important indicator of physiological status (Cole et al. 1999); however, the recovery rate also provide valuable diagnostic and prognostic information about the subject. Some authors do not explicitly calculate ΔRQ, but still use the terminology. For example, Prior et al. (Prior et al. 2014) studied the changes in RQ from a fasted state to various low‐intensity exercise sessions which they considered “metabolic flexibility during exercise”. One recent study defined MF as the change in non‐protein RQ (∆NPRQ) at the end of a refeeding diet period compared to the end of a caloric restriction diet period (Kahlhofer et al. 2014). Other researchers investigated how subjects adapt their fat oxidation to a high‐fat diet and consider this metabolic flexibility (Battaglia et al. 2012; Dube et al. 2014). Bergouignan et al. (2013) has also pointed out the variety of MF definitions and came up with another way to measure MF – by the variance of NPRQ and the variance of insulin.

Metabolic flexibility studies typically use the euglycemic‐hyperinsulinemic clamp to assess insulin sensitivity during a fasted or insulin‐stimulated state. RQ is then measured under fasting and insulin‐stimulated conditions by calorimetry (room, hood) or via blood samples, and the RQ values would be averaged over a certain period of time. Unlike the euglycemic‐hyperinsulinemic clamp, room calorimetry alone is noninvasive and allows the subject to be ambulatory. It also allows for a more physiological response, whereas in the clamp technique, insulin and glucose are infused via a catheter, and blood measurements would determine the amount of glucose to infuse from outside the body.

The primary goal of this study was to determine whether the whole‐body calorimeters can be used as tools to study short‐term changes in substrate oxidation in humans, and the secondary goal was to demonstrate that the advanced deconvolution techniques can be sensitive enough to differentiate RER response to meals with different macronutrient content and to different exercise routines. To achieve these goals, a protocol was designed to examine the subjects’ RER responses to two different nutritional and exercise challenges. The main focus of this paper is the RER response to diet.

## Materials and Methods

### Participants

The study protocol described in this paper was approved by the MedStar Health Research Institute Institutional Review Board and all volunteers provided written, informed consent. This study was registered at clinicaltrials.gov as NCT01987388.

Subjects were recruited from the area around the Beltsville Human Nutrition Research Center (BHNRC) and at the University of Maryland via fliers and emails. Volunteers were required to complete a health history questionnaire and come to the Center for a health screening. During this visit, their height, weight, and blood pressure were measured and body mass index (BMI) was calculated. The volunteers also provided a blood and urine sample to perform the following tests: lipid panel, comprehensive metabolic panel, complete blood count (CBC), thyroid‐stimulating hormone (TSH), fasting glucose, and urinalysis. Based on the lab results, it was determined whether a volunteer was eligible to participate. Of the eligible volunteers, 16 (eight men and eight women) were selected to take part in the study (Table 1). Volunteers were nonsmokers, and had no history of diabetes, cancer, or metabolic disorders.

**Table 1:** Physical characteristics of the subjects. Mean ± SEM

	Males (*n* = 8)	Females (*n* = 8)	Range
Age (years)	29 ± 2	25 ± 1^b^	22–34
BMI (kg/m^2^)	25.3 ± 1.6	24.1 ± 1.2	19.3–31.0
VO_2_peak (mL/kg/min)	43.7 ± 4.8	38.9 ± 2.1	29.3–63.6
% Body fat^a^	19.5 ± 2.8	28.2 ± 2.4^b^	9.2–37.6
Fasting glucose (mg/dL)	90 ± 2	84 ± 3	71–99
Blood pressure (mmHg)	119 ± 5	121 ± 7	100–157
	70 ± 3	70 ± 3	58–83
HOMA‐IR	1.53 ± 0.43	1.70 ± 0.39	0.47–4.49

a One female subject dropped before completing a DXA scan.

b Significantly different (*p* < 0.05).

Prior to the calorimeter stays, subjects reported to the Center to perform a VO_2_peak test. A metabolic cart (TrueOne^®^ 2400; Parvo Medics, Inc., Sandy, UT) was used to determine the oxygen consumption of the air flowing through the facemask. The test results were used to set the exercise intensity level for each participant for the calorimeter exercise protocols.

On the subjects’ third or fourth calorimeter stay, a dual‐energy X‐ray absorptiometry (DXA) scan was performed to determine their percent body fat (Hologic QDR Discovery A, Hologic, Bedford, MA).

### Study protocol

The 24‐h calorimeter protocol consisted of 2 days. On Day 1, the subjects came to the Center in the morning to consume breakfast and receive a packed lunch to take with them. Subjects were provided a base diet on Day 1 (~58% carbohydrates, 25% fat, and 17% protein). Subjects returned to the Center at ~4:15 pm. They were fitted with an ambulatory monitoring device (Equivital^™^ EQ01 LifeMonitor; Hidalgo Limited, Cambridge, UK) and a continuous glucose monitoring system (CGMS; iPro2, Medtronic MiniMed, Northridge, CA) which records subcutaneous glucose concentration. According to manufactures website, the accuracy of iPro2 is 9.9% for adults and 10.1 for children. The ambulatory monitoring device measures and records heart rate, respiration rate, skin temperature, body heat flux, activity, body core temperature, and body orientation. To measure the heat flux, two high‐resolution thermistors and a comparator circuit were used to measure the temperature difference between the front and the rear of the sensor electronics module. The heat flux devices have been compared to calibrated ceramic heat flow disks (Concept Engineering, Old Saybrook, CT) and they have been shown to have a proportional relationship. These measurements were used to correlate subject's activity levels with calorimeter variables. Both devices were worn throughout the entire stay and were removed upon exiting the calorimeter. The sampling period of the ambulatory monitoring device was 15 sec, while the continuous glucose monitor (CGM) collected data every 5 min. The volunteers entered the calorimeter room at 4:30 pm to start the 24‐h calorimeter session. They consumed dinner in the calorimeter at approximately 6 pm. They had to finish dinner by 6:45 pm to ensure that they were fasted for 12 h before their baseline blood draw the following morning. On Day 2 at ~7:15 am, the subjects received a standard breakfast of waffles and syrup, which contained 91 g of carbohydrates (Table 2). They had 15 min to consume breakfast. Blood samples were collected before and 30, 60, 90, and 120 min after breakfast and lunch to measure glucose, insulin, and nonesterified fatty acids. Homeostatic model assessment – insulin resistance (HOMA‐IR) was calculated as described in (Matthews et al. 1985).

**Table 2:** Day 2 standardized menu with macronutrient content

	Energy (kcal)	Carbohydrate (g)	Fat (g)	Protein (g)
Waffles and syrup	486	91	11	8
High‐CHO shake	548	123	3	7
High‐fat shake	519	12	51	7

Between breakfast and exercise, the subjects had personal time to browse the Internet, watch TV, or read. During each calorimeter stay, at approximately 10 am, the participants performed one of the two exercise treatments on the treadmill (Smooth Fitness 7.1HR; Smooth Fitness, Mt. Laurel, NJ) either high intensity – short duration (HI) or low intensity – long duration (LI). HI was performed at 85% of the subjects’ VO_2_peak and involved 4–5‐min sessions on the treadmill with 5 min of rest in between. LI was one 40 min session on the treadmill at 65% of the subjects’ VO_2_peak. Staff monitored the exercise sessions to record the start and stop times of the sessions and to ensure that the subjects were not struggling or having difficulty using the treadmill.

They subjects received a standard lunch at approximately 12 pm which consisted of either a high‐fat (HF) or high‐carbohydrate (HC) shake (Table 2). They were required to consume the shake in 15 min, and first and last bite times were recorded. The subjects left the calorimeter room at around 4:30 pm on day two.

During the whole study, each subject participated in four treatment combinations:

- A: low intensity – long duration/high‐fat shake
- B: high intensity – short duration/high‐fat shake
- C: low intensity – long duration/high‐carbohydrate shake
- D: high intensity – short duration/high‐carbohydrate shake

The treatment sequence was developed using two 4 × 4 Latin Squares for each gender. Subjects were then randomly assigned to a treatment sequence. Eight men and seven women completed the study. One woman dropped out of the study after completing three out of four treatments and did not complete a DXA scan. Her results for the treatments that she did complete were included in the analysis.

### Description of the calorimeter chamber

The Beltsville Human Nutrition Research Center (indirect, open‐circuit, room‐size chamber) is a “push” type calorimeter with total physical volume of 21,000 L designed to comfortably house subjects for at least 24 h while measuring the subject's respiratory gas exchange rates, energy expenditure, and respiratory quotient. Currently, BHNRC has three identical chambers each equipped with furniture, a personal computer, TV, treadmill, and a “Murphy Style” folding bed. The air is thoroughly mixed inside the chamber by a ceiling fan.

The mole fractional concentrations of nitrogen, oxygen, argon, helium, methane, and carbon dioxide in the incurrent and excurrent air are measured every 80 sec using a multiple‐gas analyzer (model MGA‐1200; Perkin‐Elmer Industrial Instruments, Pomona, CA). The data are later resampled for analysis to have 1 min sampling period. This device is a multiple collector mass spectrometer designed to measure the partial pressure of helium [2% full scale (FS)], methane (1% FS), nitrogen (100% FS), oxygen (22% FS), carbon dioxide (2% FS), and argon (2% FS)] in air within 0.1% FS. The multiple‐gas analyzer can accurately measure differences in nitrogen concentration (60.003% FS; as determined from changes in inlet air composition over 24 h and differences in inlet and outlet air composition during equilibrium), FO_2_ (60.02% FS), and FCO_2_ (60.03% FS) in chamber air so that oxygen depletion and carbon dioxide accumulation can be determined. The chamber air flow rate that is, inlet dry air flow rate at standard temperature and pressure, is measured every 5 sec using a laminar flow element (CME Vol‐O‐Flow11–25–300A; Aerospace Control Products, Davenport, IA), and a 1‐min average (typically 1.5 L/sec) is determined. The accuracy of the flow meter is 0.5% of the FS (300 L/min) according to manufacturer's specifications. The volumetric flow rate is a function of the pressure drop across the laminar flow element (Pd; electronic manometer, Datametrics, Wilmington, MA), the inlet air temperature (Ti; model RTDPR‐14–2–100, Omega Engineering, Stamford, CT), the absolute air pressure (Pi; model PX623–020A10CV, Omega Engineering), and the inlet water vapor fraction (FH2Oi; model 1200APS dew point hygrometer, General Eastern Instrumentation, Watertown, MA). A complete description of the calorimeter system is available (Seale et al. 1991) with the only major change being rewriting of the software using LabViewTM (National Instruments Corp, Austin, TX) and relocating to a new facility). Overall system performance is routinely checked by burning a known amount of pure ethanol in calorimeter chambers. The major advantage of the metabolic chamber over metabolic cart is that, provided an accurate deconvolution algorithm is deployed, it can measure minute‐by‐minute metabolic response in near‐free living conditions for 24 h or longer. Such measurements would be prohibitive with the cart due to constraints imposed on the subject.

### Concentrations’ data deconvolution using augmented regularization

The molar balance equation for gaseous exchange rates in the calorimeter can be formulated as, (Brown et al. 1984; Moon et al. 1995):

(1) $$\begin{array}{cc} R_{\mathrm{gas}}= & \left(F_{\mathrm{air}}^{\mathrm{in}}\cdot \frac{C_{N_{2}}^{\mathrm{in}}}{C_{N_{2}}^{\mathrm{out}}}\cdot C_{\mathrm{gas}}^{\mathrm{out}}\right)-\left(F_{\mathrm{air}}^{\mathrm{in}}\cdot C_{\mathrm{gas}}^{\mathrm{in}}\right)\\ & +V\frac{dC_{\mathrm{gas}}^{\mathrm{out}}}{dt}-V\cdot \frac{C_{\mathrm{gas}}^{\mathrm{out}}}{C_{N_{2}}^{\mathrm{out}}}\cdot \frac{dC_{N_{2}}^{\mathrm{out}}}{dt} \end{array}$$

where *R*_gas_ is the rate of gas production (CO_2_ and CH_4_) or gas consumption (O_2_) by the subject in the chamber in liters (L) per minute (min) (L/min). $F_{\mathrm{air}}^{\mathrm{in}}$ is the measured inlet air flow rate in L/min, $C_{\mathrm{gas}}^{\mathrm{in}}$ and $C_{\mathrm{gas}}^{\mathrm{out}}$ are the measured mole fractional concentrations of O_2_, CO_2_, or CH_4_ in the inlet and outlet air, and *V* is the volume of the chamber in liters. Both, $F_{\mathrm{air}}^{\mathrm{in}}$ and *V* have been corrected to standard temperature, pressure, and dry (STPD) conditions. The inlet $C_{N_{2}}^{\mathrm{in}}$ and outlet $C_{N_{2}}^{\mathrm{out}}$ nitrogen concentrations in Eq. (1) are given by $C_{N_{2}}^{\mathrm{in}}=1-C_{O_{2}}^{\mathrm{in}}-C_{{\mathrm{CO}}_{2}}^{\mathrm{in}}$ and $C_{N_{2}}^{\mathrm{out}}=1-C_{O_{2}}^{\mathrm{out}}-C_{CO_{2}}^{\mathrm{out}}$ The ratio $\text{HF}=\frac{C_{N_{2}}^{\mathrm{in}}}{C_{N_{2}}^{\mathrm{out}}}$ is called Haldane factor and is used to calculate incurrent or excurrent flow rates when only one of them is measured (Brown et al. 1984; Moon et al. 1995). Notice, that the rate *R*_gas_ is negative for O_2_ as the oxygen is actually consumed, not produced. Assuming that nitrogen is neither consumed nor produced by the subject in the chamber, hence its concentration's derivative is practically zero, we can neglect the last term $V\cdot \frac{C_{\mathrm{gas}}^{\mathrm{out}}}{C_{N_{2}}^{\mathrm{out}}}\cdot \frac{dC_{N_{2}}^{\mathrm{out}}}{dt}$ in the Eq. (1) thus simplifying it to:

(2) $$R_{\mathrm{gas}}=F_{\mathrm{air}}^{\mathrm{in}}\cdot \left(C_{\mathrm{gas}}^{\mathrm{out}}\cdot \text{HF}-C_{\mathrm{gas}}^{\mathrm{in}}\right)+V\frac{dC_{\mathrm{gas}}^{\mathrm{out}}}{dt}$$

and subsequently Eq. (2) is used in all our calculations. The goal of the following derivation is to demonstrate that recovering gaseous exchange rates is equivalent to solving Fredholm integral equation of the first kind. Since solving an integral equation is usually an ill‐posed problem, the regularization is applied to obtain correct solution. By rearranging the terms in Eq. (2), we can arrive to the differential equation in its standard form:

(3) $$\frac{dC_{\mathrm{gas}}^{\mathrm{out}}}{dt}+\frac{F_{\mathrm{air}}^{\mathrm{in}}\cdot \text{HF}}{V}\cdot C_{\mathrm{gas}}^{\mathrm{out}}=\frac{R_{\mathrm{gas}}}{V}+\frac{F_{\mathrm{air}}^{\mathrm{in}}}{V}\cdot C_{\mathrm{gas}}^{\mathrm{in}}$$

and putting $\frac{F_{\mathrm{air}}^{\mathrm{in}}\cdot \text{HF}}{V}=\alpha$ yields

(4) $$\frac{dC_{\mathrm{gas}}^{\mathrm{out}}}{dt}+\alpha \cdot C_{\mathrm{gas}}^{\mathrm{out}}=\frac{R_{\mathrm{gas}}}{V}+\frac{F_{\mathrm{air}}^{\mathrm{in}}}{V}\cdot C_{\mathrm{gas}}^{\mathrm{in}}$$

where the time constant of the chamber can be expressed as *T* = 1/α.

Supplying Eq. (4) with zero initial conditions we arrive to the following initial value problem:

(5) $$\frac{dC_{\mathrm{gas}}^{\mathrm{out}}}{dt}+\alpha \cdot C_{\mathrm{gas}}^{\mathrm{out}}=\frac{R_{\mathrm{gas}}}{V}+\frac{F_{\mathrm{air}}^{\mathrm{in}}}{V}\cdot C_{\mathrm{gas}}^{\mathrm{in}},C_{\mathrm{gas}}^{\mathrm{out}}\left(0\right)=C_{\mathrm{gas}}^{\mathrm{in}}\left(0\right)=0$$

This initial value problem has the following actual solution:

(6) $$V\cdot \left[C_{\mathrm{gas}}^{\mathrm{out}}-\frac{1}{V}\cdot \int_{0}^{t}e^{-\alpha (t-\tau )}\cdot F_{\mathrm{air}}^{\mathrm{in}}\left(\tau \right)\cdot C_{\mathrm{gas}}^{\mathrm{in}}\left(\tau \right)d\tau \right]=\int_{0}^{t}e^{-\alpha (t-\tau )}\cdot R_{\mathrm{gas}}\left(\tau \right)d\tau$$

where the left‐hand side represents the difference between outlet and inlet partial gas volumes and the right‐hand side is a convolution integral between chamber's impulse response e^−αt^ and the gas exchange rate *R*_gas_. Notice that the left‐hand side of Eq. (6) can be measured, while the right‐hand side contains analytically available exponential function with known decay parameter α, and the unknown part *R*_gas,_ which needs to be found. In practice, Eq. (6) has to be discretized to turn it into a system of linear equations. After discretization, the minimization problem becomes (Tokuyama et al. 2007, 2009; Gribok et al. 2013):

(7) $$argmin\left\{\|P-H\cdot R{\|}^{2}+\lambda \cdot \|L\cdot R{\|}^{2}\right\}$$

where *P* is noise‐contaminated *N *×* *1 vector of the partial volumes differentials sampled at times *t*_0_, *t*_1_, *t*_2_,…*t*_*N*−1_, H is a lower triangular *N*×*N* matrix of calorimeter room's impulse response function, *R* is an *N *×* *1 vector of unknown gas exchange rates sampled at times *t*_0_, *t*_1_, *t*_2_,…*t*_*N*−1_. The trade‐off parameter λ is the regularization parameter controlling the degree of smoothness of the regularized solution, while *L* is an *N* − 2 × *N* band matrix of discrete approximation of the second‐order derivative. The ∥·∥ denotes Euclidean norm. To be used in Eq. (7), the *P* vector was resampled to 1 min intervals. Notice that expression (7) is a functional, that is it maps solution vector *R* into a number.

For this paper, the following algorithm was used to recover gaseous exchange rates and RER from concentrations measurements:

1. The CO_2_ production rate: $R_{{\mathrm{CO}}_{2}}$ was obtained by solving the minimization problem represented by functional 7. The regularization parameter $\lambda_{{\mathrm{CO}}_{2}}$ was selected using the discrepancy principle (Morozov 1993) with the upper bound on the noise variance in CO_2_ concentrations obtained from the previously collected vacant rooms’ data.
2. Having calculated the CO_2_ production rate, we used it as an a priori estimate for the O_2_ consumption rate, by including it into minimization algorithm via functional 8. Functional 8 was minimized with respect to $R_{O_{2}}$.

(8) $$argmin\left\{\|P-H\cdot R_{O_{2}}{\|}^{2}+\lambda \cdot \|L\cdot \left(R_{O_{2}}-R_{CO_{2}}\right){\|}^{2}\right\}$$

1. Having obtained gaseous exchange rates, RER is calculated as $R_{{\mathrm{CO}}_{2}}/R_{O_{2}}$.

Notice that functional 8 is minimized for $R_{O_{2}}$ while $R_{{\mathrm{CO}}_{2}}$ is used as an *a priory* estimate constraining further the range of possible solutions for $R_{O_{2}}$. The use of $R_{{\mathrm{CO}}_{2}}$ as an a priori estimate for the $R_{O_{2}}$ is motivated by the fact that under aerobic metabolism, the two signals are highly correlated as increase in one causes corresponding increase in the other. However, since out of the two signals, the $R_{{\mathrm{CO}}_{2}}$ is the more accurate one, it can be used as an additional constraint for its nosier counterpart ‐ $R_{O_{2}}$. As was pointed out above, the lower accuracy in $R_{O_{2}}$ is especially detrimental for RER calculations as $R_{O_{2}}$ is in the denominator and its small values can greatly affect the RER's estimate.

The analysis of the penalty term in Eq. (8) reveals that minimizing the second‐order derivatives of the difference between two functions, we maximize their correlation. The regularization parameter $\lambda_{O_{2}}$ for $R_{O_{2}}$ can be selected either by applying the discrepancy principle or by setting $\lambda_{O_{2}}$ to match the correlation between two signals obtained from a more accurate device, for example, a metabolic cart. During aerobic respiration, $R_{O_{2}}$ and $R_{{\mathrm{CO}}_{2}}$ are highly correlated because increase in consumption inevitably causes a corresponding increase in production. For this study, since accurate minute‐to‐minute gaseous exchange rates were obtained for each subject during VO_2_peak tests, those exchange rates were used to calculate the correlation coefficient, and the regularization parameter $\lambda_{O_{2}}$ was set to match the correlation coefficient obtained on the metabolic cart data collected while subjects were metabolizing aerobically. For the high‐intensity exercise sessions, the regularization parameter was selected using the discrepancy principle since the subjects were most likely exercised at levels above their gas exchange thresholds and the correlation coefficient did not reflect the relationships between gaseous exchange rates.

The performance of the deconvolution algorithm was previously described in detail in (Tokuyama et al. 2007, 2009; Gribok et al. 2013).

## Results

**Figure 1:**
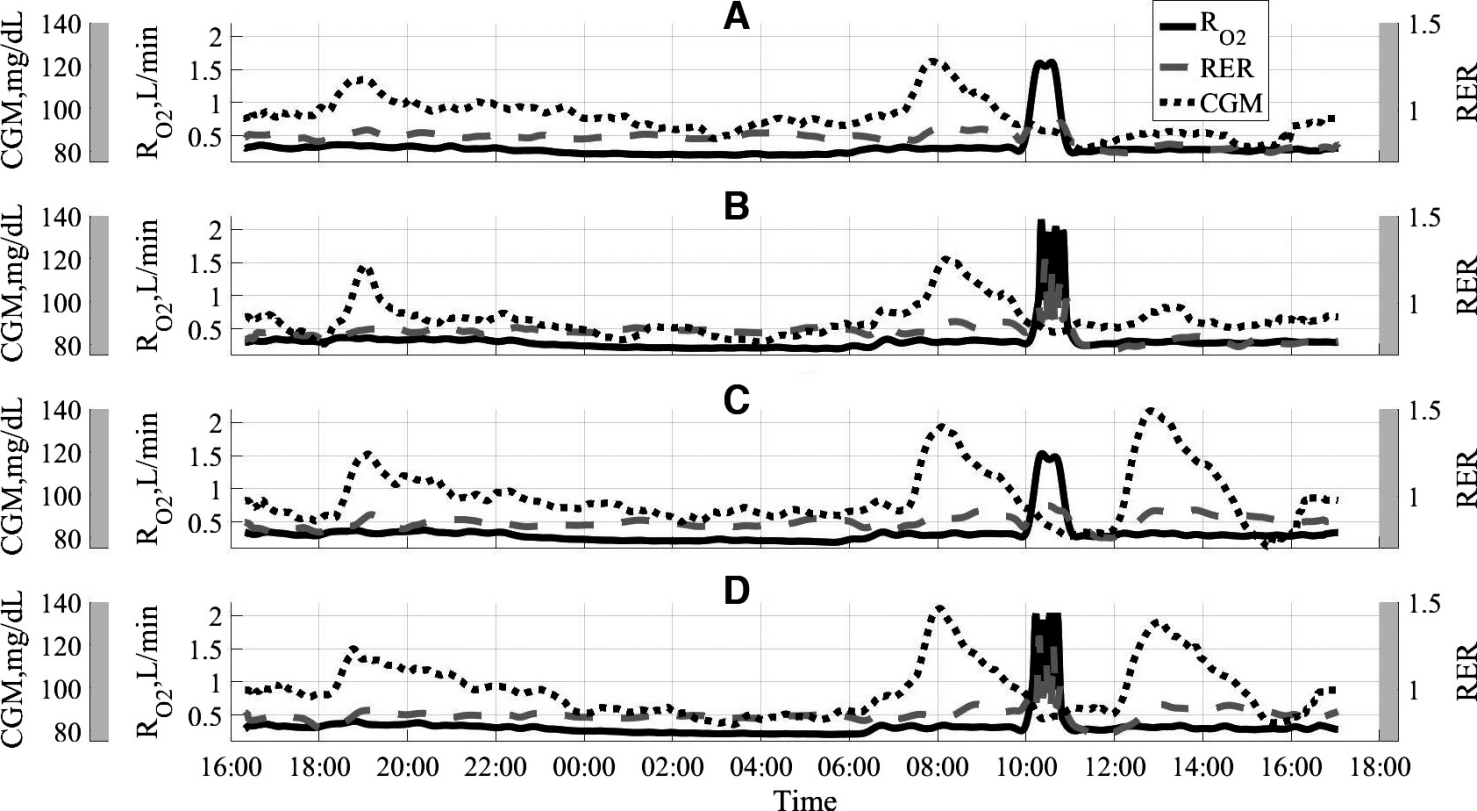
Concurrent time series data for four treatment combinations used in the study. A‐low intensity/high fat, B‐high intensity/high fat, C‐low intensity/high carb, D‐high intensity/high carb.

Figure 1 shows concurrent 24‐h time series data for oxygen consumption‐$R_{O_{2}}$, subcutaneous glucose concentrations‐CGM, and respiratory exchange ratio‐RER. The curves represent averaged over 16 sessions (15 in case of treatment combination D) time series for each of the four treatment combinations. The top panel in Figure 1 shows the concurrent data for treatment combination A when the low‐intensity long‐duration exercise was combined with a high‐fat shake at lunch. Panel B (second from the top) shows high‐fat shake combination with high‐intensity short‐duration exercise. The bottom two panels, C and D, show high‐carbohydrate shake combined with low‐intensity and high‐intensity exercise sessions, respectively. All time series data were resampled to 1 min sampling period using a combination of linear interpolation and anti‐aliasing filter.

**Figure 2:**
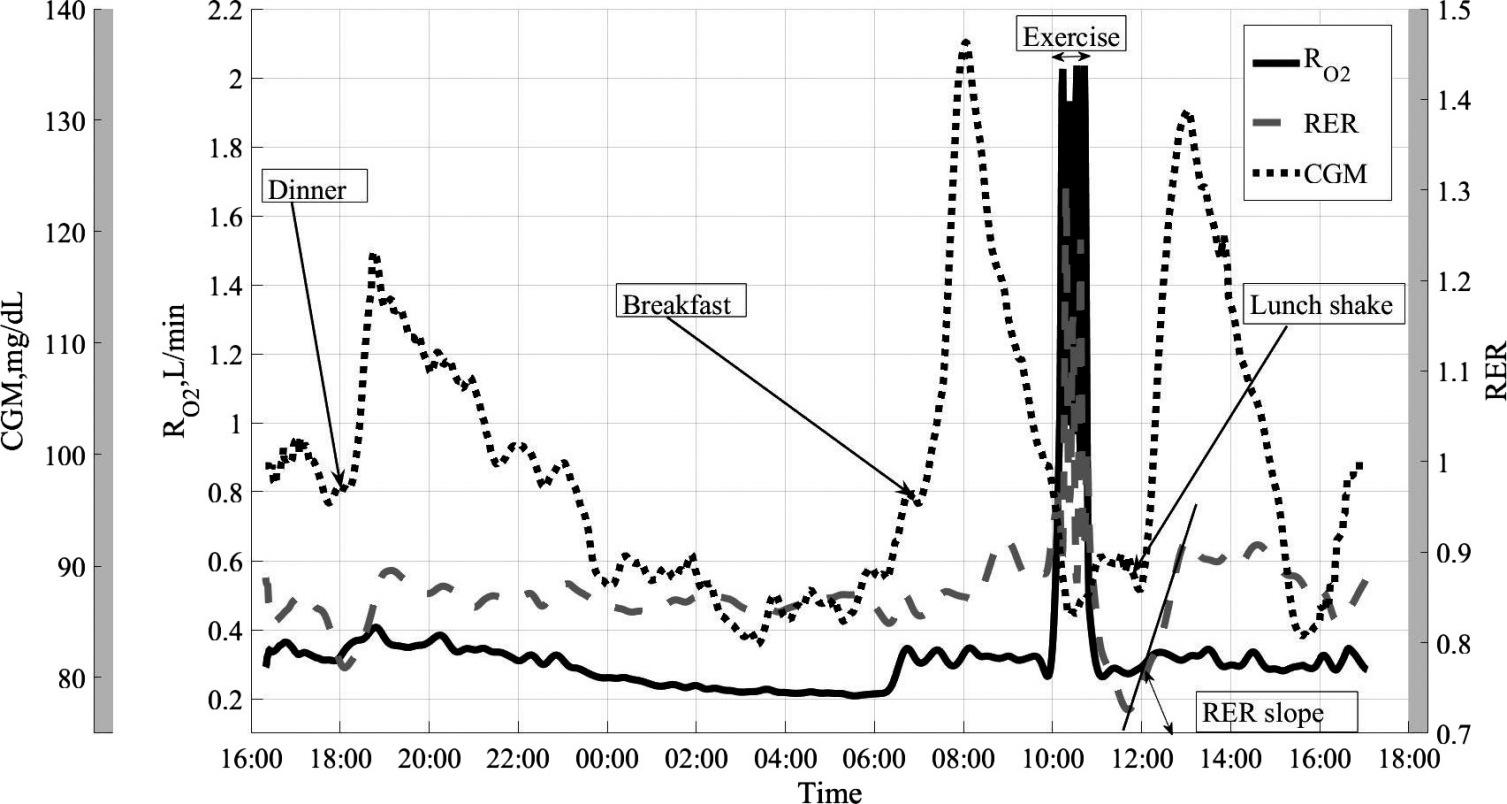
Concurrent data for treatment combination D, high intensity/high carb.

Figure 2 shows concurrent time series data for treatment combination D along with time stamps for meals and exercise activity. Also, the RER postprandial slope is indicated as a mean to quantify post meal substrate oxidation response.

**Figure 3:**
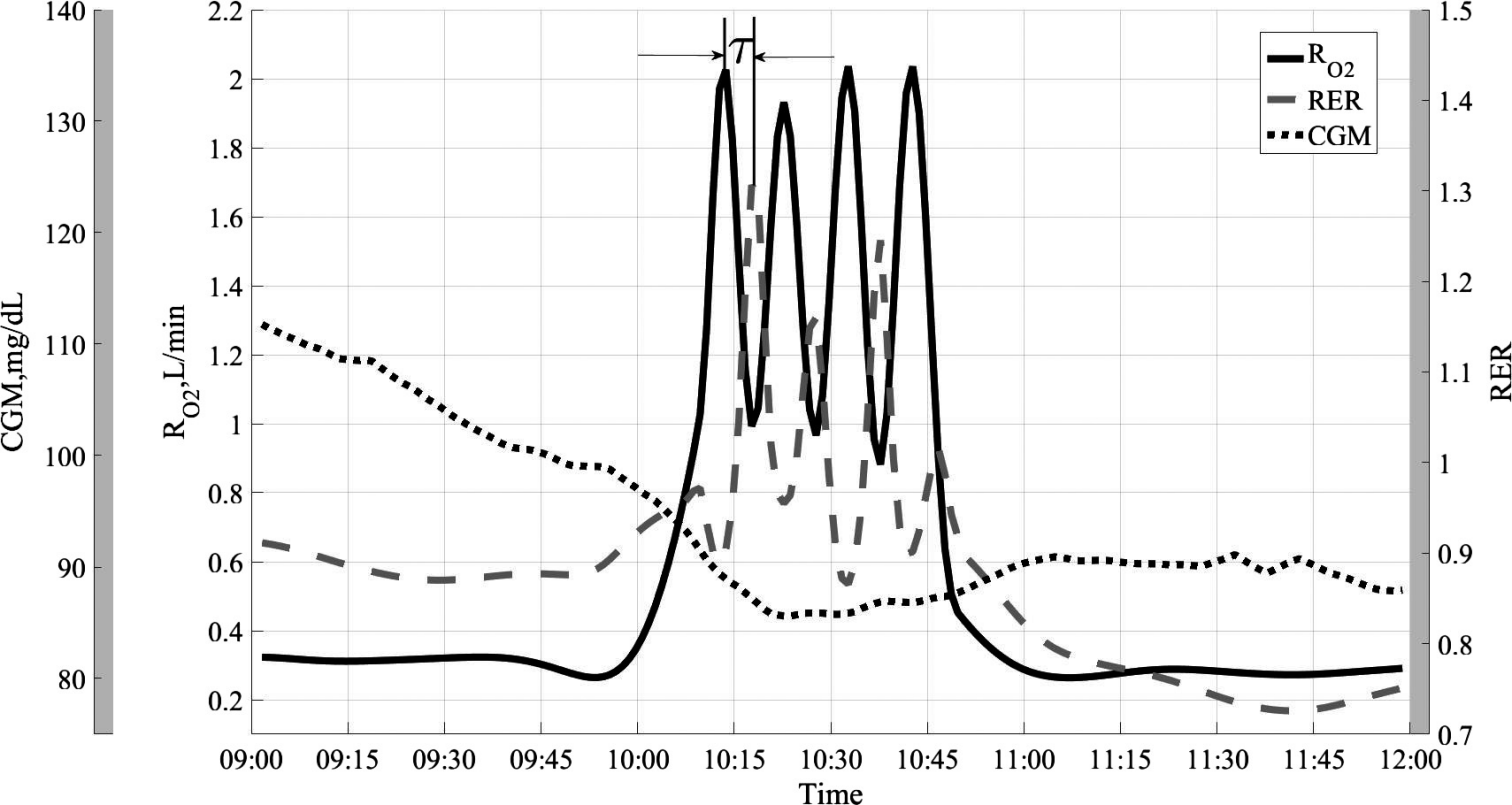
Exercise period for treatment combination D, high intensity/high carb.

Figure 3 shows the exercise period for treatment combination D. The time lag τ between the peak in oxygen consumption and peak in RER is typical for high‐intensity exercises and is due to excess postexercise CO_2_ production.

**Figure 4:**
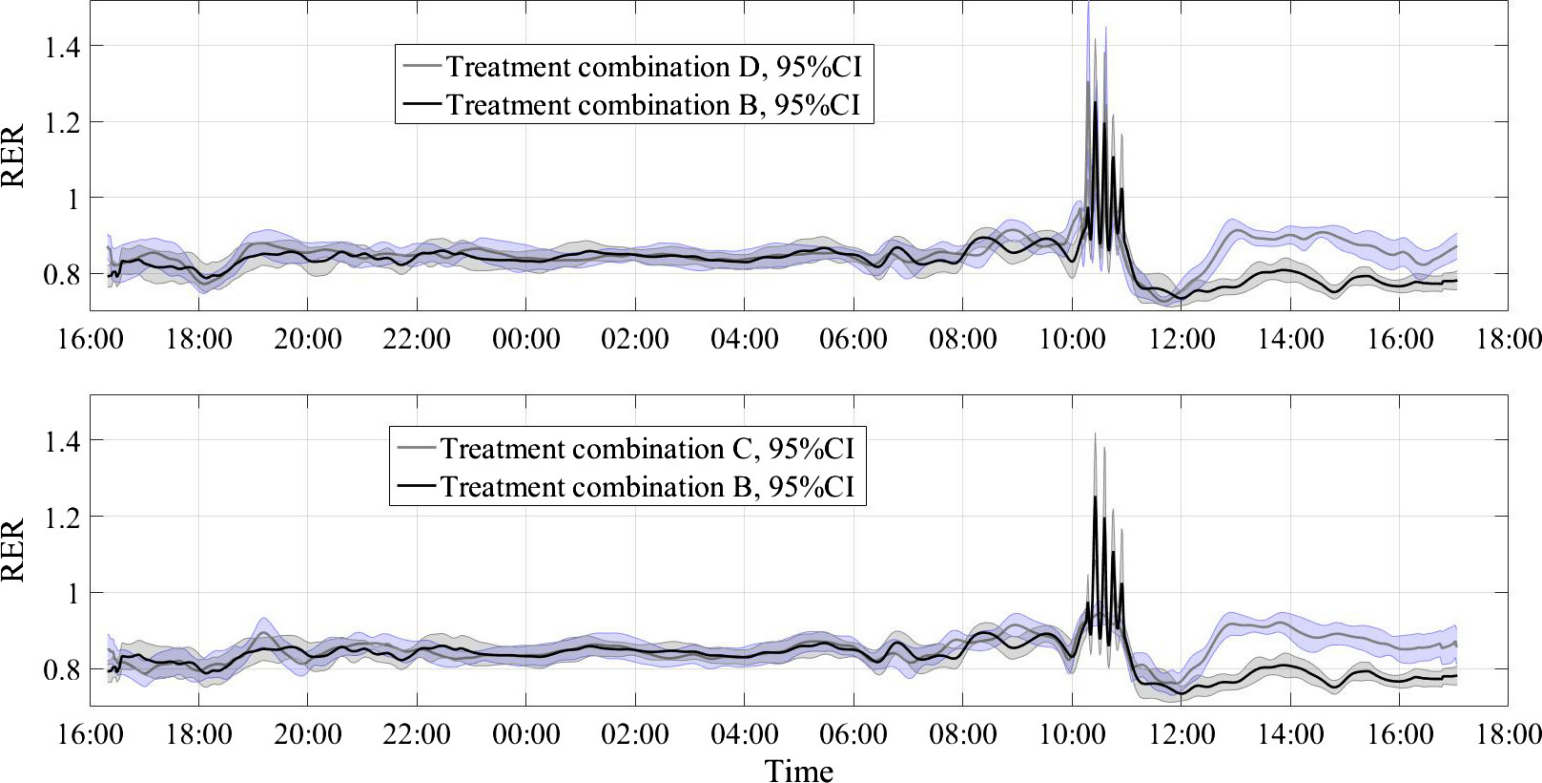
Respiratory exchange ratio (RER) values along with 95% pointwise confidence intervals (CI). B‐high intensity/high fat, C‐low intensity/high carb, D‐high intensity/high carb.

**Figure 5:**
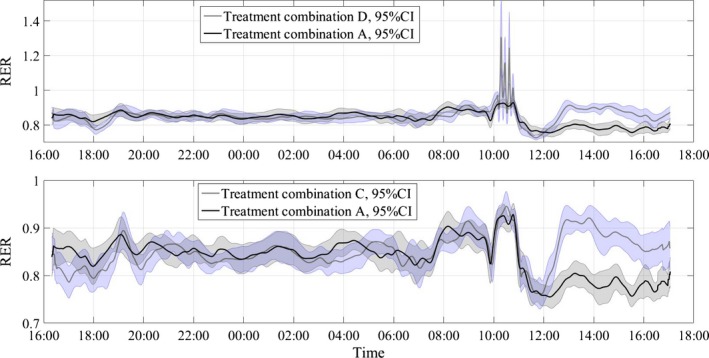
Respiratory exchange ratio (RER) values along with 95% pointwise confidence intervals (CI). A‐low intensity/high fat, C‐low intensity/high carb, D‐high intensity/high carb.

**Figure 6:**
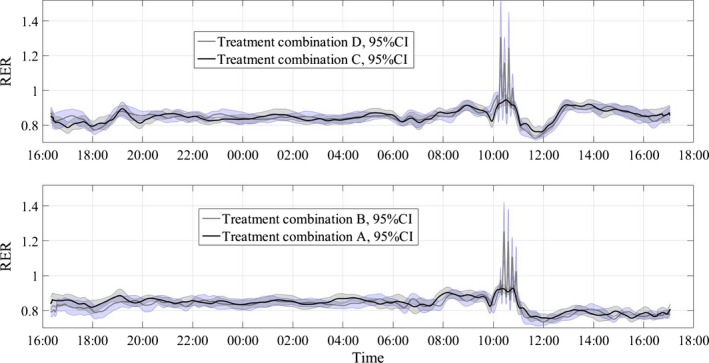
Respiratory exchange ratio (RER) values along with 95% pointwise confidence intervals (CI). A‐low intensity/high fat, B‐high intensity/high fat, C‐low intensity/high carb, D‐high intensity/high carb.

**Figure 7:**
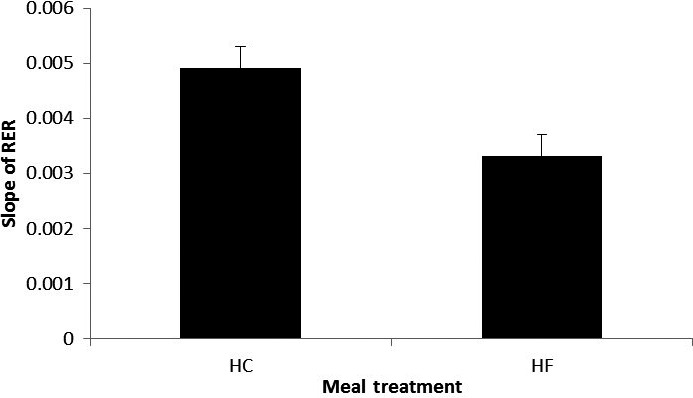
Slope of RER following the treatment lunch. *p* = 0.0059; HC, high carbohydrate; HF, high fat, RER, respiratory exchange ratio. The error bars are the standard errors of the means. The units of the RER's slope is changes in RER per minute.

Figures 4, 5, 6 show the average RER time series for different treatment combinations along with 95% pointwise confidence intervals calculated as mean value ± *t*(1 − α/2; *n* − 1)·SE(mean value), where the *t*(1 − α/2; *n* − 1) is the percentile of the Student distribution with *n* − 1 degrees of freedom, and SE is the standard error of the mean value calculated as s/√*n* with *s* being sample standard deviation around the mean value. For this study, the value of *n* = 16 for all treatment combinations but D, where *n* = 15, due to a subject's dropout. These confidence intervals are pointwise, not simultaneous, and can only be used to test statistically significant difference between the average curves not intracurve variability. The 95% pointwise confidence intervals are shown as shaded bands around the average curves. Figure 7 shows average values for postprandial RER slope (0.0049 (SE = 0.0004) vs. 0.0033 (SE = 0.0004); *p* = 0.0059) along with standard error bars. The RER slope was calculated separately for each calorimeter session. The mean values of the slope were estimated for high‐carbohydrate and high‐fat shakes regardless of the exercise intensity, since there was no significant shake*exercise interaction. Thus, the slope values at Figure 7 are the averages over 32 and 31 sessions with corresponding error bars. The high‐carbohydrate average is calculated over 31 trials since one subject did not perform the D treatment combination. The slope was calculated the way it is shown in Figure 2. Linear least squares regression was fitted to the RER data starting at 12:00 pm and ending at the maximum RER value after lunch. The dependent or paired‐samples t‐test was used to check for differences in RER slopes.

## Discussion

For many decades, indirect whole‐body respirometry has been the method of choice to study energy expenditure and substrate oxidation in humans. The majority of these studies reported daily averages for such metabolic variables as energy expenditure and respiratory exchange ratio. While the daily averages undoubtedly provide valuable physiological information, they do not provide insights into temporal variability, rate of change, and frequency content of metabolic variables. On the other hand, the short‐term physiological measurements have recently enjoyed an unprecedented level of success and technological advancements. Heart rate variability, continuous glucose monitoring, and core body temperature telemetry pills are just a few examples of such short‐term measurements which are clinically useful and are available as wearable modalities. However, there is a paucity of studies that have examined and presented short‐term substrate oxidation response to meals in humans. One notable exception is (Treuth et al. 2003) where authors present and analyze hourly average RERs throughout the 24‐h calorimetry study after consumption of the low‐fat, high‐carbohydrate and high‐fat, low‐carbohydrate meals. However, the data are 1‐hour mean values and no pointwise statistical analysis was performed on time series data to demonstrate that two meal treatments can be discerned. The goal of this paper is to address this lack of experimental material and provide data and evidence that the whole‐body calorimeter can be used to study short‐term substrate utilization in humans under different exercise and dietary treatments.

Figure 1 shows the summary of the time series data for all four treatment combinations. It is clear that the HC lunch meal treatments produced significant elevation in subcutaneous glucose concentrations, as expected. The rise in glucose concentration after HC lunch is comparable for both HI and LI exercises. Also, since breakfast contained a significant amount of carbohydrates, the CGM response after this meal is comparable with the response to HC lunch treatment. The breakfast macronutrient content was identical for all four treatment combinations (Table 2), which produced similar post meal response shown in Figure 1 with subcutaneous glucose concentration peaking around 8:00 am.

To demonstrate the concurrent dynamics of the three time series in more detail, Figure 2 shows the RER, CGM, and oxygen consumption rate for only one treatment combination‐D. This treatment combination included high‐intensity exercise and high‐carbohydrate lunch. Postprandial elevations in glucose and RER are clearly visible. Notice, as subjects woke up at around 6:15–6:30 am, their glucose levels started to rise along with oxygen production. Consumption of breakfast significantly accelerated the rise in glucose concentration. Figure 2 also shows a well‐coordinated concurrent rise between postprandial CGM and RER responses. It is also clear that the CGM response to meals is significantly more acute than RER as CGM recovery rate is higher than RER. For example, after HC lunch shake, the glucose concentration reaches its maximum and starts to decrease within an hour, while RER stays elevated for few more hours and starts to fall off at approximately at 3:00 pm. By that time, glucose concentration essentially reached its baseline. The temporal dynamics of RER is especially important as it allows estimating such important variables as RER's rate of change and its correlation with glucose concentration. The post lunch RER response shows that it takes about an hour for substrate oxidation to change from the baseline value of ~0.83 to predominantly carbohydrates utilization with RER = 0.91. Having reached the maximum value, the RER then remained plateaued for approximately two hours. The subjects were asked to consume the lunch shakes within 15 min, however, it rarely took more than 2 min, and thus the HC lunch can be considered as a nutritional carbohydrate delta function and its response represent dynamical characteristics of the system.

Figure 3 shows detailed response to HI exercise challenge for treatment combination D. This is a zoomed‐in part of exercise in Figure 2. The four 5‐min of HI exercise bouts are clearly visible along with four peaks in RER. The RER and $R_{O_{2}}$ time series are perfectly negatively correlated during the exercise. The time lag τ between two signals is due to excess postexercise carbon dioxide production, which is caused by lactic acid buffering by sodium bicarbonate (Wasserman et al. 1973; Beaver et al. 1986; Yunoki et al. 1999; Peronnet and Aguilaniu 2006; Yano et al. 2009). The exercise rate at 85% of VO_2_peak is above the lactate threshold for most individuals and this increase in lactate concentration causes reduction in bicarbonate concentration in the blood, which in turn causes elevated CO_2_ production (Wasserman et al. 1973). After the exercise, the produced lactic acid is buffered to CO_2_ causing the continuous rise in CO_2_ production rate, while the oxygen consumption starts to fall. Since RER = $R_{{\mathrm{CO}}_{2}}$/$R_{O_{2}}$, the concurrent drop in denominator and rise in the numerator lead to higher RER values. The drop in RER at the beginning of the exercise at around 10:13 am is attributed to reported (Linnarsson 1974; Hughson and Inman 1985) CO_2_ storage at the onset of constant load exercise. Also, of note is a continuous downward trend in subcutaneous glucose concentration which can be attributed to usage of muscle glycogen during intensive exercise.

Figures 4, 5, 6 present the main results reported in this paper. The upper panel in Figure 4 shows the difference in dietary treatment response for combinations B and D. Both treatment combinations included high‐intensity exercise with a high‐fat or high‐carbohydrate lunch treatments, respectively. Prior to those lunch treatments, both combinations are identical in terms of exercise and dietary conditions. After 12:00 pm, the RER response for two treatment combinations starts to diverge with significantly higher RER values for combination D, which included high‐carbohydrate lunch shake. The shaded areas around the curves are the 95% pointwise confidence intervals and they cease to overlap at around 12:30 pm indicating that the two curves are pointwise statistically different from that time on with *p* < 0.01 (Cumming et al. 2007). For the remainder of the calorimeter session, the 95% confidence intervals are widely separated indicating that the two curves are statistically different with *p *≪ 0.01 (Cumming et al. 2007). The bottom panel in Figure 4 illustrates substrate oxidation response for treatment combinations C and B. The treatment combination C included low‐intensity exercise and high‐carb lunch treatment. An important observation is that the metabolic response to high‐carb meal is very similar to the treatment combination D, which demonstrates that substrate oxidation response does not depend on the intensity of the preceding exercise. It is also obvious that the two curves stayed statistically different for the rest of the calorimeter session. Figure 5 shows concurrent substrate oxidation response for treatment combinations D and A, as well as for C and D. Notice, the *Y*‐axis scale on the bottom panel is different from the top panel. The top panel demonstrates that the exercise intensity prior to the meal treatment does not affect the substrate oxidation response, while the bottom panel shows that the RER values are statistically different when two different meal treatments are administered after low‐intensity exercise. Notice that the substrate utilization remained statistically different for 4 h after the meal treatments. Figure 6 is presented to demonstrate consistency of the described approach with respect to dietary responses. The top panel shows RER time series for treatment combinations C and D. The only difference between these two combinations is the exercise intensity; the meal treatments are identical. Aside from the exercise period, the 95% pointwise confidence intervals significantly overlap indicating that the two curves are statistically identical and the utilized calculation technique produce consistent repeatable results. The bottom panel in Figure 6 demonstrates the same degree of repeatability for high‐fat treatments combined either with high‐ or low‐intensity exercise. Figure 7 summarizes statistical information on the differences between metabolic responses to two lunch treatments. The RER slopes, shown in Figure 2, for two types of dietary responses were calculated for each calorimeter session. The average slope for each dietary treatment regardless of the intensity of the preceding exercise was calculated and the average values along with standard error bars are plotted. It can be seen that the slopes are statistically different, demonstrating the sensitivity of the approach to different macronutrient contents.

The current results indicate that minute‐to‐minute substrate oxidation response can be effectively measured in whole‐body indirect calorimeter. The results of this study also demonstrate that the minute‐to‐minute measurements of substrate oxidation in the whole‐body calorimeter can be performed with statistically meaningful consistency. One of the definitions of metabolic flexibility (Sparks et al. 2009b) is “the capacity for skeletal muscle to acutely shift its reliance between lipids and glucose during fasting or in response to insulin, such as in postprandial conditions”; however, very few published results convincingly demonstrated that the “acute” response to dietary challenges can be effectively measured in the whole‐body calorimeter, yet the whole‐body respirometry remains the “gold standard” technique for metabolic measurements in humans. The ability to measure short‐term substrate oxidation response is essential to understanding obesity, diabetes, and metabolic disorders. The ability to measure such response in the whole‐body calorimeter also significantly broadens the spectra of available tools and allows conducting studies on metabolic response under nearly free leaving conditions. Additionally, proliferation of hand held and wearable physiological sensors along with the arrival of implantable devices raised the problem of their thorough validation under free living conditions. The majority of the new wearable devices target whole‐body short‐term energy expenditure and metabolism as one of their major output variables and highly accurate minute‐to‐minute calorimeter room measurements may provide a much needed test bed under free living conditions.

## Disclaimer

The opinions or assertions contained herein are the private views of the author(s) and are not to be construed as official or as reflecting the views of the Army or Department of Defense.

## Conflict of Interest

None declared.
